# A Pan-Cancer Analysis of Prognostic and Immunological Roles for Cell Death Genes

**DOI:** 10.3390/genes14061178

**Published:** 2023-05-28

**Authors:** Ye Hong, Yan Yuan, Zekun Liu, Zexian Liu, Yizhuo Zhang

**Affiliations:** 1 State Key Laboratory of Oncology in South China, Collaborative Innovation Center for Cancer Medicine, Sun Yat-sen University Cancer Center, Guangzhou 510060, China; hongye@sysucc.org.cn (Y.H.); yuanyan9128@163.com (Y.Y.); liuzk@sysucc.org.cn (Z.L.); 2 Department of Pediatric Oncology, Sun Yat-sen University Cancer Center, Guangzhou 510060, China

**Keywords:** pan-cancer, cell death, prognosis, TME

## Abstract

The dysregulation of cell death is closely associated with the development, progression, tumor microenvironment (TME), and prognosis of cancer. However, there is no study that comprehensively explores the prognostic and immunological role of cell death in human pan-cancer. We used published human pan-cancer RNA-sequencing and clinical data to explore the prognostic and immunological roles of programmed cell death, which included apoptosis, autophagy, ferroptosis, necroptosis, and pyroptosis. A total of 9925 patients were included for bioinformatic analysis, with 6949 and 2976 patients in the training cohort and validation cohort, respectively. Five-hundred and ninety-nine genes were defined as programmed-cell-death-related genes. In the training cohort, 75 genes were identified to define PAGscore by survival analysis. According to the median value of PAGscore, patients were divided into high- and low-risk groups, and subsequent analyses demonstrated that the high-risk group had a higher level of genomic mutation frequency, hypoxia score, immuneScore, expression of immune genes, activity of malignant signaling pathways, and cancer immunity cycle. Most anti-tumor and pro-tumor components of the TME showed greater activity in high-risk patients. Scores of malignant cell properties were also higher in high-risk patients. These findings were confirmed in the validation cohort and external cohort. Our study constructed a reliable gene signature to distinguish prognosis-favorable and prognosis-unfavorable patients and demonstrated that cell death was significantly associated with cancer prognosis and the TME.

## 1. Introduction

Malignancies are the main threat to human health and an important obstacle to the extension of life expectancy of the population [1]. The occurrence of cancer is increasing with the aging of the population and pollution of the environment [2,3]. In 2020, an estimated 19.3 million new cancer diagnoses and about 10 million cancer deaths occurred worldwide. Moreover, the worldwide cancer burden is expected to rise by 47% by 2040 compared with 2020 [4]. Although multimodal therapies such as surgery, chemotherapy, and radiotherapy have been taken, most patients show poor prognosis [5]. Exploring the mechanisms of development and progression of cancers to reduce incidence, identify precise therapeutic targets, and improve outcomes is still urgent.

Cell death is an important process that maintains human body homeostasis by regulating cell proliferation and the stress response [6,7]. The dysregulation of cell death is closely associated with cancers. Several studies have revealed that dysregulated cell death would promote neoplasia, cause cancer drug resistance, and influence the cancer prognosis [8,9,10]. Apoptosis, autophagy, ferroptosis, necroptosis, and pyroptosis are the most extensively investigated cell death types in the cancer field [11,12,13,14,15,16]. Loss of apoptosis is generally considered to be a cause of cancers, and apoptosis-inducing agents are widely developed and used to treat cancers [8,17]. The roles of the other four types of cell death in cancer are controversial, with some studies reporting their tumor-promoting effects and others reporting their anti-tumor effects [10,18,19]. Additionally, many studies have also demonstrated that those five types of cell death could affect the tumor microenvironment (TME) in different cancers [20,21,22,23]. However, what we should pay attention to is that most studies report the role of one type of cell death in one certain cancer. There is no study that comprehensively evaluates the prognostic and immunological role of those five types of cell death in human pan-cancer.

In the present study, we explored the prognostic and immunological role of five types of cell death in human pan-cancer by bioinformatic analysis and constructed a cell death signature, namely prognosis-associated gene score (PAGscore). The PAGscore of each patient was calculated based on the expression of prognosis-associated genes and their corresponding coefficients of the multivariate Cox regression analysis as follows: PAGscore = Coef1 * Exp1 + Coef2 * Exp2 + …… + Coef75 * Exp75. We demonstrated that PAGscore significantly correlated with the clinical characteristics and previously reported gene signatures. Moreover, we also revealed that PAGscore was closely associated with TME. Our findings indicated the potential connection between cell death, TME, and the prognosis of pan-cancer.

## 2. Materials and Methods

### 2.1. Data Collection and Preprocessing

We downloaded human pan-cancer-normalized and batch-removed RNA-sequencing data and the corresponding clinical data from the UCSC Xena portal (UCSC Xena (xenabrowser.net)), and excluded lymphoma and leukemia samples for subsequent analysis. Samples were divided into the training group and validation group according to the random ratio of 7:3 by using the R package “caret” [24]. A liver cancer cohort from the International Cancer Genome Consortium (ICGC) was used as the external cohort to validate the findings of the present study. The genomic mutation data were obtained and analyzed by the R package “TCGAbiolinks” [25] and “maftools” [26]. Meanwhile, sequencing data were also downloaded from the Gene Expression Omnibus (GEO) database by using the R package “GEOquery” [27] to explore and validate gene signatures of five types of cell death.

### 2.2. Establishment of PAGscore

We obtained signaling pathways of apoptosis, autophagy, ferroptosis, necroptosis, and pyroptosis from the KEGG database [28], Reactome pathway knowledgebase [29], and WikiPathways database [30]. All genes of these signaling pathways were extracted and defined as cell-death-related genes. The univariate and multivariate Cox regression analyses and LASSO analyses were applied to explore the prognosis-prediction role of cell-death-related genes in the training cohort. We calculated the PAGscore of each patient based on the expression of genes and their corresponding coefficients of multivariate Cox regression analysis as follows: PAGscore = Coef1 * Exp1 + Coef2 * Exp2 + …… + Coef75 * Exp75. All inclusion samples were eventually divided into high- and low-risk groups based on the median value of PAGscore.

### 2.3. Construction of Cell Death Gene Signatures

To construct unique gene signatures for apoptosis, autophagy, ferroptosis, necroptosis, and pyroptosis, we first extracted genes from corresponding cell-death-signaling pathways and then curated positive-regulated genes of each kind of cell death according to the literature. Each cell death signature was constituted by the corresponding positive regulated genes. In addition, we obtained datasets that compare the RNA-sequencing data of control cells with death-induced cells in the GEO database (Home—GEO—NCBI (nih.gov)) to test the performance of our cell death signatures. 

### 2.4. Evaluation of the Clinical and Molecular Characteristics of High- and Low-Risk Groups

To further investigate the underlying mechanisms of survival difference between high- and low-risk patients, we compared the age, gender, tumor mutation burden (TMB), neoantigen load, and mutation spectrum of fifty of the most commonly mutated genes in cancer between two groups. Fifty of the most commonly mutated genes of cancer were obtained from the literature [31]. We also evaluated published tumor-prognosis-associated signatures such as hypoxia score [32] and cytolytic score [33] in two groups. We used the R package “progeny” to explore the activity of 14 kinds of cancer-related pathways in two groups. Meanwhile, gene sets of fifty kinds of cancer hallmarks were downloaded from the MSigDB database (GSEA|MsigDB (gsea-msigdb.org, Accessed on 1 September 2021) and single-sample gene enrichment analysis (ssGSEA) was conducted to calculate scores of hallmarks of each patient to characterize high- and low-risk patients.

### 2.5. Comparison of TME between High-Risk and Low-Risk Patients

We obtained immunomodulators and immune checkpoint genes from the literature, and the compared method was used to calculate the “ImmuneScore”, “StromaScore”, and “TumorPurity” of the two groups of patients [34,35,36]. We obtained gene sets of 29 components of the TME [37] and 12 gene sets of the cancer immunity cycle [38] from the literature and used the R package “GSVA” [39] to calculate the score of those gene sets for each patient to comprehensively compare the difference in TME between the two groups of patients.

### 2.6. Statistical Analysis

All statistical analyses were implemented in R 4.05 software (https://www.r-project.org/, accessed on 1 September 2021). The Wilcoxon–Mann–Whitney test and Kruskal–Wallis test were used to compare non-normal-distribution continuous variables between two groups and more than two groups, respectively. The chi-square test was applied to compare categorical variables. Pearson correlation analysis was used to explore the correlation between variables. Significant correlations were defined as Pearson coefficients greater than 0.2 in absolute terms and *p* < 0.05. The Kaplan–Meier method was used to plot survival curves for patients, and the log-rank test was used to determine statistically significant differences. For univariate Cox regression analysis, we used *p* value ≤ 0.001 as the cutoff value to select significantly survival-associated genes. Except for the univariate Cox regression analysis, *p* value < 0.05 was considered statistically significant.

## 3. Results

### 3.1. Construction of Gene Signatures for Programmed Cell Death

The present study included 9925 cancer patients with obtainable transcriptome data and clinical data. According to a ratio of 7 vs. 3, all patients were grouped into the training group of 6949 cases and the validation group of 2976 cases. 

**Figure 1:**
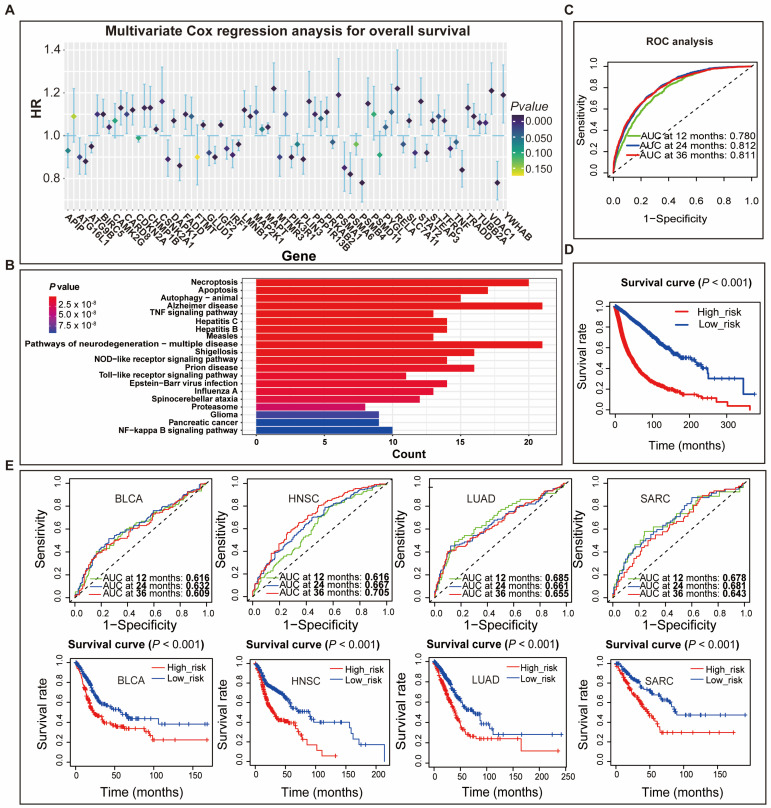
Constructing and validating overall-survival-associated programmed-cell-death signature—PAGscore. (**A**) Multivariate Cox regression analysis identified 75 programmed-cell-death-related genes constructing the best-predicting model for overall survival in the training cohort; (**B**) Enriched analysis of KEGG pathways of 75 programmed-cell-death-related genes. (**C**) The ROC analysis showed that PAGscore could effectively predict the 12-, 24-, and 36-month overall survival rate for patients in the training cohort; (**D**) The survival analysis showed that the overall survival of high-risk patients with higher PAGscore was worse than that of low-risk patients with a lower PAGscore in the training cohort; (**E**) The results of ROC and survival analyses showed that PAGscore performed well in predicting the overall survival in several types of cancer patients. Abbreviations: HR, hazard ratio; CI, confidential interval; BLCA, bladder urothelial carcinoma; HNSC, head and neck squamous cell carcinoma; LUAD, lung adenocarcinoma; SARC, sarcoma.

Signaling pathways of apoptosis, autophagy, ferroptosis, necroptosis, and pyroptosis were downloaded from the Kyoto Encyclopedia of Genes and Genomes (KEGG) database, Reactome pathway knowledgebase, and WikiPathways database. The numbers of signaling pathways of apoptosis, autophagy, ferroptosis, necroptosis, and pyroptosis were 3, 3, 2, 3, and 1, respectively (Table 1). A total of 599 genes were extracted from these signaling pathways and defined as cell-death-related genes. Through univariate Cox regression analysis, 324 genes were shown to be significantly associated with overall survival (OS) in the training cohort (Supplementary Table S1). Then, 107 survival-related genes were identified by the LASSO Cox regression analysis (Supplementary Figure S1A,B). Finally, multivariate Cox regression analysis identified 75 independent prognostic-related genes that could be used to construct the optimal prognostic model (Figure 1A). We also used the R function “enrichKEGG” to explore the enriched cell death pathway and found that the pathways of necroptosis, apoptosis, and autophagy-animal were significantly enriched (Figure 1B). The individual PAGscore was calculated for each patient based on the expression levels of 75 genes and the coefficients of multivariate Cox regression analysis. ROC analysis showed that the AUCs of PAGscore in predicting 12-, 24- and 36-month survival were 0.78, 0.812, and 0.811, respectively, which suggested that PAGscore was a reliable prognosis predictor (Figure 1C). Based on the median PAGscore, patients were divided into high- (higher PAGscore) and low-risk groups (lower PAGscore). Survival analysis revealed a poorer prognosis for the high-risk group than the low-risk group (Figure 1D). PAGscore also performed well in predicting the survival of patients in the validation cohort. The AUCs of PAGscore in predicting survival at 12, 24, and 36 months were 0.76, 0.779, and 0.771, respectively (Supplementary Figure S1C). Survival analysis showed that low-risk patients had a better prognosis (Supplementary Figure S1D). Furthermore, PAGscore also effectively predicted the survival of patients of a certain type of cancer, such as lung adenocarcinoma (LUAD), head and neck squamous cancer (HNSC), sarcoma (SARC), and bladder urothelial carcinoma (BLCA) (Figure 1E, Supplementary Figures S2 and S3).

For each type of cell death, we merged the genes of the corresponding signaling pathways and extracted the positively regulated genes of cell-death as the cell death signature. Eventually, signatures of apoptosis, autophagy, ferroptosis, necroptosis, and pyroptosis included twenty-five, eleven, twenty-eight, fourteen, and eleven genes, respectively (Table 1). The “ssGSEA” method was used to calculate the scores of five types of cell death for each patient based on the above signatures’ gene sets. The numbers of GEO datasets used to verify the performance of cell death signatures of apoptosis, necroptosis, autophagy, ferroptosis, and pyroptosis were thirteen, three, fourteen, six, and two, respectively (Table 1). Our results demonstrated that five types of cell death signatures could all greatly distinguish control cells and death-induced cells (Supplementary Figures S4 and S5). 

### 3.2. The Characteristics of Five Types of Cell Death Gene Signatures in Cancers

**Figure 2:**
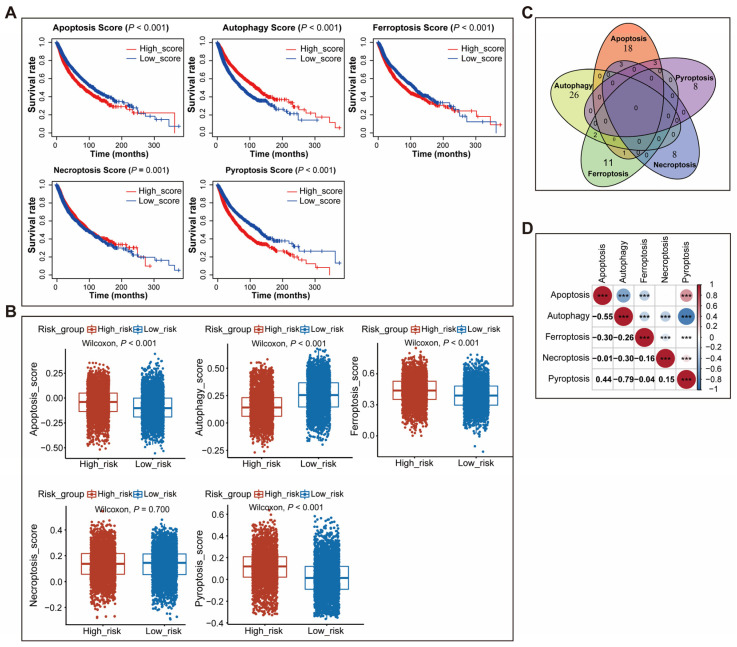
Identifying and characterizing five types of cell death gene signatures. (**A**) The survival analysis showed that apoptosis score, ferroptosis score, and pyroptosis were unfavorable survival predictors, but autophagy score and necroptosis score were favorable survival predictors in the training cohort; (**B**) The box plots showed that high-risk patients had higher scores of apoptosis, ferroptosis, and pyroptosis, and a lower score of autophagy than low-risk patients; (**C**) The Venn diagram showed that the genes of five types of cell death signatures had a few overlaps; (**D**) Correlation analysis of five types of cell death signatures scores in pan-cancer patients. ***: *p* < 0.001.

To determine the prognostic significance of five types of cell death gene signatures, survival analysis was proceeded in the two cohorts and the results showed that autophagy score was a prognosis-favorable factor; however, ferroptosis score and pyroptosis score were prognosis-unfavorable factors (Figure 2A, Supplementary Figure S6A). Apoptosis score was negatively associated with prognosis in the training cohort, but not correlated to prognosis in the validation cohort. Necroptosis score was significantly positively associated with prognosis in the training cohort, but only showed a non-significant positive trend in the validation group. Comparing the five types of cell death signature scores between two different risk-group patients revealed that high-risk patients had a lower autophagy score and a higher apoptosis score, ferroptosis score, and pyroptosis score in both cohorts (Figure 2B, Supplementary Figure S6B).

To further explore the association between five types of cell death signatures, we first analyzed the overlap between those signatures’ genes. The Venn diagram showed that the number of overlapping genes among different signatures was small (Figure 2C). Next, Pearson correlation analysis was performed to evaluate the correlation between each score of the two signatures in human pan-cancer. The results demonstrated that apoptosis score was negatively associated with autophagy score (Pearson coefficient = −0.55, *p* < 0.001) and ferroptosis score (Pearson coefficient = −0.3, *p* < 0.001), and positively associated with pyroptosis score (Pearson coefficient = 0.44, *p* < 0.001); autophagy score was negatively related to ferroptosis score (Pearson coefficient = −0.26, *p* < 0.001), necroptosis score (Pearson coefficient = −0.3, *p* < 0.001), and pyroptosis score (Pearson coefficient = −0.79, *p* < 0.001) (Figure 2D).

We also evaluated the association between the scores of the five signatures in a certain type of cancer, such as breast cancer, glioblastoma, and esophageal carcinoma. Consistent with the results of pan-cancer, apoptosis score was negatively related to autophagy score and ferroptosis score and positively associated with pyroptosis score; autophagy score was negatively related to ferroptosis score, necroptosis score, and pyroptosis score in several kinds of cancers (Supplementary Figure S7).

### 3.3. Associations between PAGscore Groupings and Clinical and Molecular Features of Cancers

**Figure 3:**
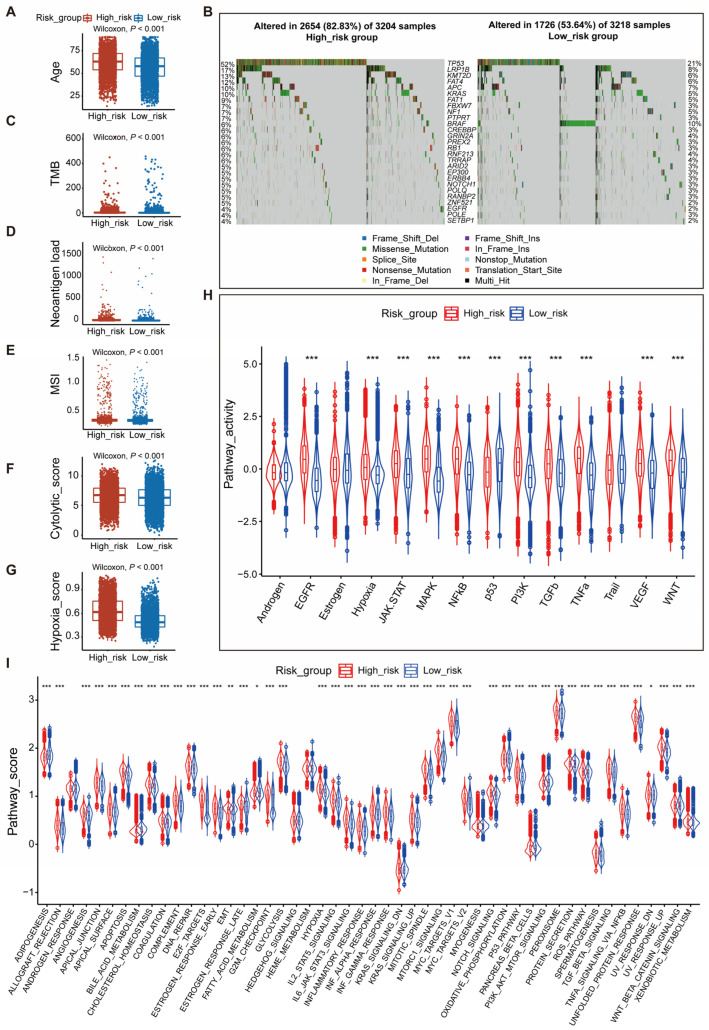
Comparing the clinical and molecular features between high- and low-risk patients in the training cohort. (**A**) The box plot showed that high-risk patients have an older-age distribution than low-risk patients; (**B**) Analysis of the mutation profiles of the fifty most commonly mutated genes in human cancers showing that 27 genes had a significantly different mutation frequency between low-risk and high-risk patients; (**C**) The box plot showed that high-risk patients have a higher TMB than low-risk patients; (**D**) The box plot showed that high-risk patients have a higher neoantigen load than low-risk patients; (**E**) The box plot showed that high-risk patients have a higher MSI than low-risk patients; (**F**) The box plot showed that high-risk patients have a higher cytolytic score than low-risk patients; (**G**) The box plot showed that high-risk patients have a higher hypoxia score than low-risk patients; (**H**) The relative signaling pathway activity in high- and low- risk patients was evaluated by the R package “progeny”; (**I**) The violin plot showed the ssGSEA score of fifty pathways of cancer hallmarks in high- and low-risk patients. Abbreviations: TMB, tumor mutation burden; MSI, microsatellite instability. *: *p* < 0.05, **: *p* < 0.01, ***: *p* < 0.001.

To characterize high- and low-risk groups, we comprehensively compared the clinical and molecular characteristics of two groups of patients. First, we compared the age distribution of two groups. The results showed that high-risk patients were older (*p* < 0.001) (Figure 3A, Supplementary Figure S8A).

Next, we chose the fifty most frequently mutated genes in human cancers and explored the mutation profiles of the 50 genes between the two risk groups. In the training cohort, the mutation frequencies of the high- and low-risk group were 93.88% (3008/3204) and 78.03% (2511/3218) (*p* < 0.001), respectively. The high-risk group had higher mutation frequencies, and twenty-seven genes had statistical significance in both the training and validation group. Of 27 genes, *TP53* (52%), *LRP1B* (17%), and *KMT2D* (13%) were the three most mutated genes in the high-risk group, while *TP53* (21%), *BRAF* (10%), and *LRP1B* (8%) were the top three mutation genes in the low-risk group (Figure 3B). The mutation profiles of high- and low-risk groups in the validation cohort were consistent with the training cohort: the mutation frequency of the high-risk group was higher than the frequency in the low-risk group (92.39% vs. 78.21%, *p* < 0.001). *TP53* (48%), *LRP1B* (15%), and *KMT2D* (13%) were the top 3 mutation genes in the high-risk group, while *TP53* (22%), *BRAF* (10%), and *LRP1B* (7%) were the top three mutation genes in the low-risk group (Supplementary Figure S8B). Meanwhile, we also compared the TMB, neoantigen load, and microsatellite instability (MSI) between the high- and low-risk patients. Both training and validation cohorts were consistent in that high-risk patients had a higher TMB, neoantigen load, and MSI than low-risk patients (*p* < 0.001) (Figure 3C–E, Supplementary Figure S8C–E).

Finally, we analyzed the distribution of previously reported cancer-associated molecular characteristics in high-risk and low-risk groups. The results showed that the high-risk group had a higher cytolytic score (*p* < 0.001) and hypoxia score than the low-risk group (*p* < 0.001) in both training and validation cohorts (Figure 3F,G, Supplementary Figure S8F,G). In addition, malignant signaling pathway activities were assessed by using the “progeny” R package in two groups. We found that the activity of the p53 signaling pathway was higher in the low-risk group, while the activity of other pathways was higher in the high-risk group, such as EGFR, hypoxia, JAK-STAT, MAPK, NF-κB, PI3K, TGFβ, TNFα, VEGF, and WNT pathways (Figure 3H, Supplementary Figure S8H). The analysis of ssGSEA scores for fifty cancer hallmarks showed that multiple hallmarks had a higher activity in the high-risk group, for example, angiogenesis, DNA-repair, epithelial–mesenchymal transition (EMT), and hypoxia (Figure 3I, Supplementary Figure S8I).

### 3.4. Differences in TME between High- and Low-Risk Patients

To identify the immunological characteristics of the TME in high- and low-risk patients, we analyzed the ESTIMATE score, the expression of immunomodulators and immune checkpoint genes, the activity of the cancer immunity cycle, and the ssGSEA scores of 29 components of the TME.

**Figure 4:**
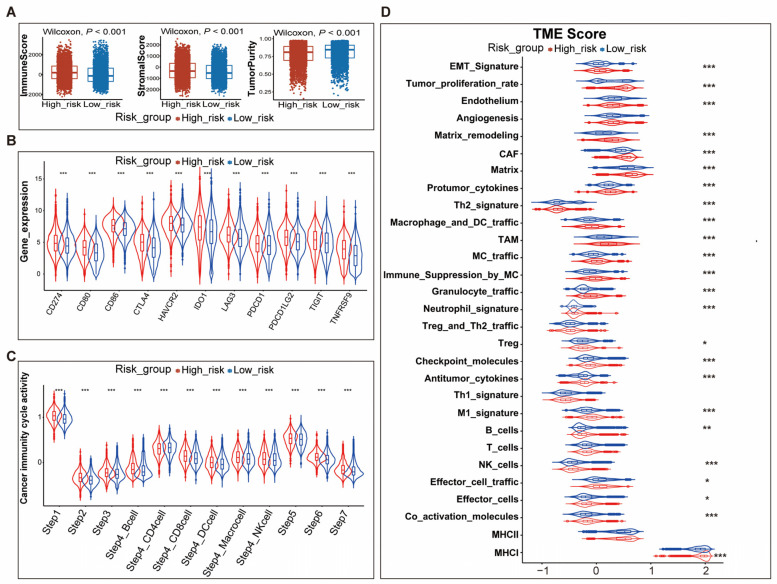
Comparing the differences in TME between high- and low-risk patients in training cohort. (**A**) The box plots showed that high-risk patients had a higher “ImmuneScore” and “StromaScore” and lower “TumorPurity” than low-risk patients; (**B**) The violin plot showed the expression of immune checkpoint genes in high- and low-risk patients; (**C**) The relative activity of seven steps of the cancer immunity cycle in high- and low-risk patients; (**D**) The violin plot showed the ssGSEA score of 29 components of TME in high- and low-risk patients. Abbreviations: TME, tumor microenvironment; CAF, cancer-associated fibroblasts; TAM, tumor-associated macrophages; MC, myeloid cells. *: *p* < 0.05, **: *p* < 0.01, ***: *p* < 0.001.

Compared to the patients in the low-risk group, high-risk patients had a higher “ImmuneScore” and “StromaScore” and lower “TumorPurity” (Figure 4A, Supplementary Figure S9A). Expression analysis of immune-related genes showed that the majority of MHC-I and MHC-II components such as HLA-A, HLA-B, HLA-C, HLA-DMB, HLA-DQA1, and HLA-DRA were up-regulated in the high-risk group, indicating that the ability of antigen presentation and processing was enhanced in patients of this group. Meanwhile, some key chemokines and corresponding receptors, including CCL5, CXCL9, CXCL13, CCR5, and CXCR3, were also significantly up-regulated in high-risk patients (Supplementary Figure S10A,B). Much solid evidence supported the role of these chemokines in promoting the recruitment of immune cells such as CD8+T cells and antigen-presenting cells. Immune checkpoint genes were also a signature of an inflamed TME and our results revealed that all immune checkpoint genes, covering CD274, CD80, CD86, CTLA4, HAVCR2, IDO1, LAG3, PDCD1, PDCD1LG2, TIGIT, and TNFRSF9, were significantly down-regulated in low-risk patients (Figure 4C, Supplementary Figure S9B).

Analysis of the activity of the cancer immunity cycle demonstrated that high-risk patients possessed a higher activity in all steps of the cancer immunity cycle, which includes seven steps. The first step was the release of cancer cell antigens and the second was the presentation of cancer antigens. The third step was the priming and activation of cancer antigens. The fourth step was the trafficking of immune cells into tumors. Then, the immune cells would infiltrate into the tumors (Step 5) and the T cells would recognize the cancer cells (Step 6). Finally, the immune cells would kill the cancer cells (Step 7) (Figure 4D, Supplementary Figure S9C).

Comprehensively evaluating the TME profile of high- and low-risk patients by calculating the ssGSEA score of 29 components of the TME suggested that components of either the anti-tumor microenvironment or pro-tumor microenvironment were generally up-regulated in high-risk patients. Malignant cell properties including proliferation rate signature and EMT signature were also up-regulated in high-risk patients. Moreover, consistent with other scores, the score of angiogenesis and fibrosis signatures in the high-risk group was higher than that of the low-risk group (Figure 4E, Supplementary Figure S9D).

In summary, these findings demonstrated that the tumor immune environment had significant differences among the high-risk patients and low-risk patients. The complex interaction between TME components ultimately determined the prognosis of cancer patients.

### 3.5. ICGC Liver Cancer Cohort Validated Study Findings

**Figure 5:**
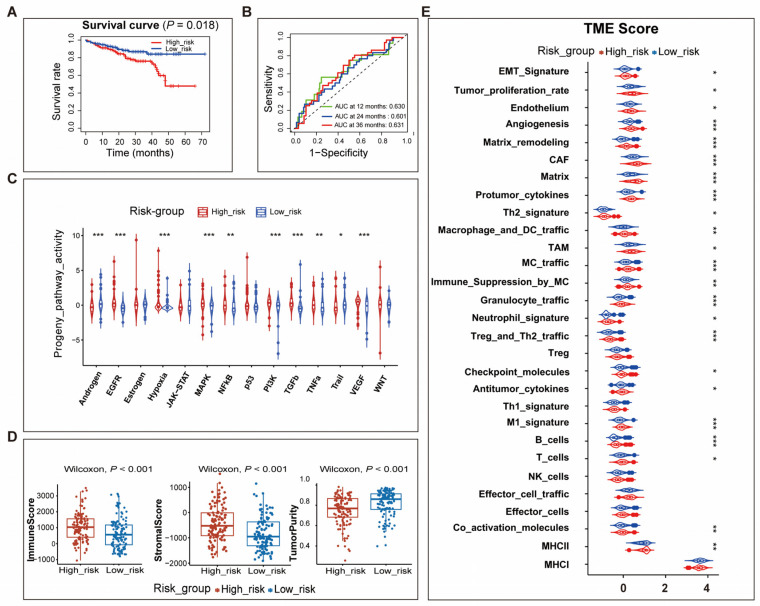
The ICGC liver cancer cohort validated the study findings. (**A**) Survival analysis showed that the overall survival of high-risk patients with a higher PAGscore was worse than that of low-risk patients with a lower PAGscore in the ICGC cohort; (**B**) The results of ROC and survival analyses showed that PAGscore performed well in predicting the overall survival in liver cancer patients; (**C**) The relative signaling pathway activity in high- and low- risk patients was evaluated by the R package “progeny”; (**D**) The box plots showed that high-risk patients had a higher “ImmuneScore” and “StromaScore” and lower “TumorPurity” than low-risk patients; (**E**) The violin plot showed the ssGSEA score of 29 components of TME in high- and low-risk patients. Abbreviations: TME, tumor microenvironment; CAF, cancer-associated fibroblasts; TAM, tumor-associated macrophages; MC, myeloid cells. *: *p* < 0.05, **: *p* < 0.01, ***: *p* < 0.001.

An ICGC liver cancer cohort with 232 patients was used as the external cohort to further validate our findings. Consistent with the training and validation cohort, PAGscore was an unfavorable prognosis factor. According to the median value of PAGscore, patients were divided into high- and low-risk groups, and subsequent analyses demonstrated that the high-risk group had a higher immuneScore and activity of malignant signaling pathways. Most anti-tumor and pro-tumor components of the TME showed greater activity in high-risk patients. Scores of malignant cell properties were also higher in high-risk patients (Figure 5).

## 4. Discussion

To our knowledge, this is the first time a study has comprehensively explored the prognostic and immunological role of five types of cell death (apoptosis, autophagy, ferroptosis, necroptosis, and pyroptosis) in human pan-cancer. Here, we defined a reliable score named PAGscore based on the prognosis-associated cell-death-related genes that could effectively distinguish prognosis-favorable and prognosis-unfavorable cancer patients. Further analyses characterizing the patients with different prognoses included the differences in clinical and molecular characteristics, the malignant potential of cancer cells, and the complexity of interaction between TME components.

We constructed PAGscore by analyzing five types of cell-death-related genes, and based on the median PAGscore, patients were split into high-risk and low-risk groups with significantly different prognoses. To clarify the impact of each type of cell death on the prognosis of patients, we also constructed the unique signature of apoptosis, autophagy, ferroptosis, necroptosis, and pyroptosis. Survival analyses showed that autophagy score was the favorable prognosis factor, while ferroptosis score and pyroptosis score were the unfavorable prognosis factors, in both training and validation cohorts. The survival effects of apoptosis score and necroptosis score were inconsistent between the training and validation cohort. We speculated that the reason for this phenomenon might be the difference in the number of patients between the two cohorts. Further comparing the distribution of scores of five cell death signatures in high- and low-risk groups, the results revealed that high-risk patients had a higher unfavorable prognosis-associated score (apoptosis score, ferroptosis score, and pyroptosis score) and lower favorable prognosis-associated score (autophagy score). It is well known that cell death plays an important role in cancer processes. As the tumor is a mixture of cancer cells and non-cancer cells, simply exploring the role of cell death by analyzing bulk tissues’ RNA-sequencing data is not accurate enough. We used the ESTIMATE method to assess the proportion of cancer cells and non-cancer cells in the TME. The results showed that high-risk patients had a higher proportion of immune cells and stromal cells, and lower tumor purity. Our cell death signature score reflected the overall cell death profile of the tumor microenvironment, which could not differentiate cancer cells and non-cancer cells. It was reported that the apoptosis, ferroptosis, and pyroptosis of immune cells could cause cancer immune evasion [40,41,42,43]. Excessive autophagy could cause cancer cell death (autophagy-dependent cell death, ADCD) [44]. In addition, there is evidence that autophagy could regulate the survival and memory formation of cytotoxic T cells [45,46,47]. Combined with our results, we speculated that the poor prognostic effects of the apoptosis score, ferroptosis score, and pyroptosis score were due to a higher proportion of immunes cells with death in the high-risk patients, while the favorable prognostic role of autophagy score was due to a higher proportion of cancer cells with ADCD in the low-risk patients.

Several studies have suggested that age and gender are important risk factors of the incidence and mortality of cancer [48,49]. We compared the age and gender distribution between high- and low-risk patients and found that the high-risk group possessed a higher proportion of older and male patients. Mutations also played a fundamental factor in the tumor development and prognosis of cancer [31]. Our results suggested that high-risk patients had higher mutation frequencies than low-risk patients. Consistent with previous reports, *TP53* was the most frequently mutated gene in both high-risk (48–52%) and low-risk patients (21%–22%) in this study, and the frequency of the *TP53* mutation was also significantly positively correlated with the risk. We also observed that the activity of the p53 pathway was higher in low-risk patients than in high-risk patients by using the R package “progeny”. *TP53* is a well-known tumor-suppressor, while the p53 inactivation caused by *TP53* mutations can escape tumor cell death and rapid tumor progression [50]. Accordingly, we concluded that the difference in prognosis between the two groups of patients was significantly associated with *TP53* mutation. In addition to a difference in tumor suppressor pathway, many pathways related to carcinogenesis and progression showed higher activity in high-risk patients, such as the EGFR pathway, MAPK pathway, and PI3K pathway. All of our findings could support the poor prognostic performance in the high-risk group.

The TME is constituted by tumor cells and non-tumor cells. The complex interaction between components of the TME determines the cancer prognosis. Our results showed that high-risk patients had an inflammatory TME relative to low-risk patients. The pro- and anti-tumor components were significantly upregulated in high-risk patients. Although the tumor purity was lower in the high-risk group than the low-risk group, the scores of protumor cytokines and tumor proliferation rate were significantly higher than those of the low-risk group. Previous studies have reported that tumor cells have the ability to dominate the microenvironment, which gives rise to the hypothesis that tumor cells recruit large numbers of surrounding cells and make them form a protective barrier [51]. Accordingly, we speculated that highly malignant cancer cells in the high-risk group promoted the formation of a unique TME and led to a poor prognosis.

The main advantage of our study was the use of a large pan-cancer cohort and the comprehensive analysis of the prognosis and immunological role of five types of cell death. Due to the limitation of bulk RNA-sequencing, we could not effectively evaluate the role of five types of cell death of different cells on the TME and prognosis of cancers. We look forward to large pan-cancer single-cell sequencing to explore the effects of different types of cell death on the TME and prognosis.

Taken together, our study found that cell-death-related genes were significantly associated with prognosis. The potential mechanisms of different prognoses between high- and low-risk patients included differences in clinical and molecular characteristics, the malignant potential of tumor cells, and the complex interplay between TME components.

## Figures and Tables

**Table 1:** The gene sets of five types of programmed cell death signatures and corresponding GEO datasets for validation.

Variables	Apoptosis	Necroptosis	Autophagy	Ferroptosis	Pyroptosis
Number of pathways	3	3	3	2	1
Reactome pathway knowledgebase (genes)	181	159	151	0	27
KEGG database (genes)	136	62	32	41	0
WikiPathways database (genes)	87	9	30	40	0
Total genes	307	163	160	41	27
Promoting cell death genes	25	11	15	14	11
Number of GEO datasets for validation	13	3	14	6	2
Number of GEO datasets with statistical significance	7	1	8	4	0
Number of GEO datasets with differential trends	6	2	6	2	2

Abbreviations: KEGG, Kyoto Encyclopedia of Genes and Genomes; GEO, Gene Expression Omnibus.

## Data Availability

The corresponding author or first author will provide data supporting this research study upon reasonable request.

## Supplementary Materials

The following supporting information can be downloaded at: https://www.mdpi.com/article/10.3390/genes14061178/s1, Figure S1: Constructing and validating overall-survival-associated programmed-cell-death signature—PAGscore; Figure S2: ROC analysis of survival-predictive performance of PAGscore in different human cancers; Figure S3. Survival analysis of high- and low-risk patients in different human cancers; Figure S4. Validating the performance of five types of cell death gene signatures in differentiating death-inducing and non-death-inducing cells; Figure S5. Validating the performance of five types of cell death gene signatures in differentiating death-inducing and non-death-inducing cells; Figure S6. Identifying and characterizing five types of cell death gene signatures in cancers; Figure S7. Correlation analysis of score of five types of cell death signatures in different types of cancers; Figure S8. Comparing the clinical and molecular features between high- and low-risk patients in validation cohort; Figure S9. Comparing the differences in tumor microenvironment between high- and low-risk patients in validation cohort; Figure S10. The expression of immune-related genes in high- and low-risk patients; Table S1: Univariate Cox regression analysis for overall survival in training cohort.
