# Supplementary material for: A Pan-Cancer Analysis of Prognostic and Immunological Roles for Cell Death Genes
Source: Genes (Basel). 2023 May 28;14(6):1178. doi: 10.3390/genes14061178 (PMC10298235; doi:10.3390/genes14061178)
Supplement: Supplementary file 1 [file genes-14-01178-s001.zip › genes-2390908-supplementary.pdf]

Supplementary Material

Pan-cancer Analysis of Prognostic and Immunological Roles for Cell Death Genes

Ye Hong, Yan Yuan, Zekun Liu, Ze-Xian Liu, Yi-Zhuo Zhang

* **Correspondence:** Prof. Yi-Zhuo Zhang, Department of Pediatric Oncology, Sun Yat-sen University Cancer Center. 651 Dongfeng East Road, Guangzhou, Guangdong 510060, P. R. China. Tel: +86-20-87342466; Fax: +86-20-87343598; Email: zhangyzh@sysucc.org.cn; Prof. Ze-Xian Liu, State Key Laboratory of Oncology in South China, Collaborative Innovation Center for Cancer Medicine, Sun Yat-sen University Cancer Center, Guangzhou 510060, P. R. China. Tel: +86-20-87342466; Fax: +86-20-87343598; Email: liuzx@sysucc.org.cn.

1 Supplementary Figures and Tables

1.1 Supplementary Figures

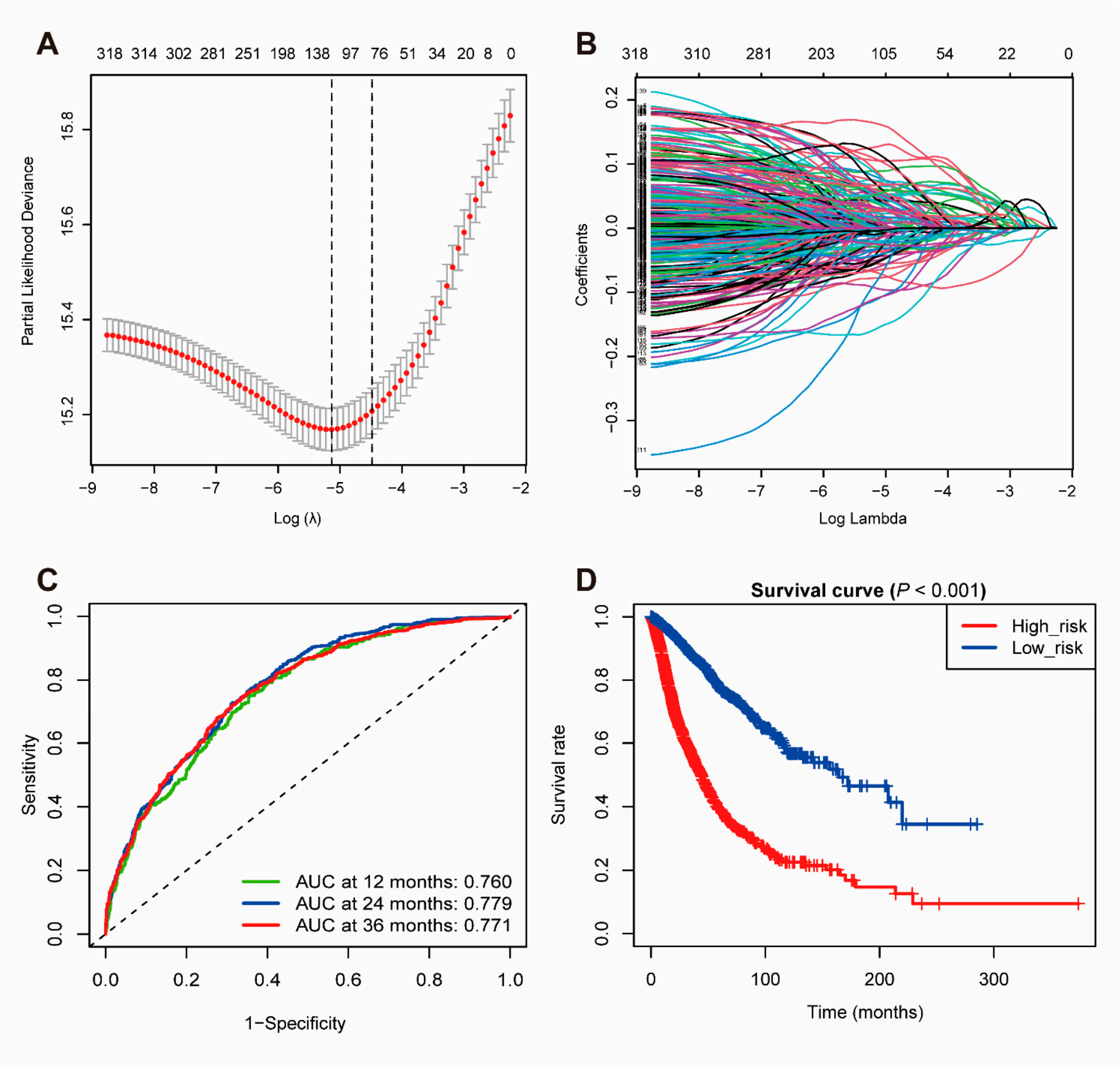

Supplementary Figure S1. Constructing and validating of overall survival associated programmed cell death signature - PAGscore. A, B) LASSO analysis showed 107 genes were most survival-related genes; C) ROC analysis showed PAGscore could effectively predict the 12-, 24- and 36-month overall survival rate of patients in validation cohort; D) Survival analysis showed the overall survival of high-risk patients with higher PAGscore was worse than low risk patients with lower PAGscore in validation cohort.

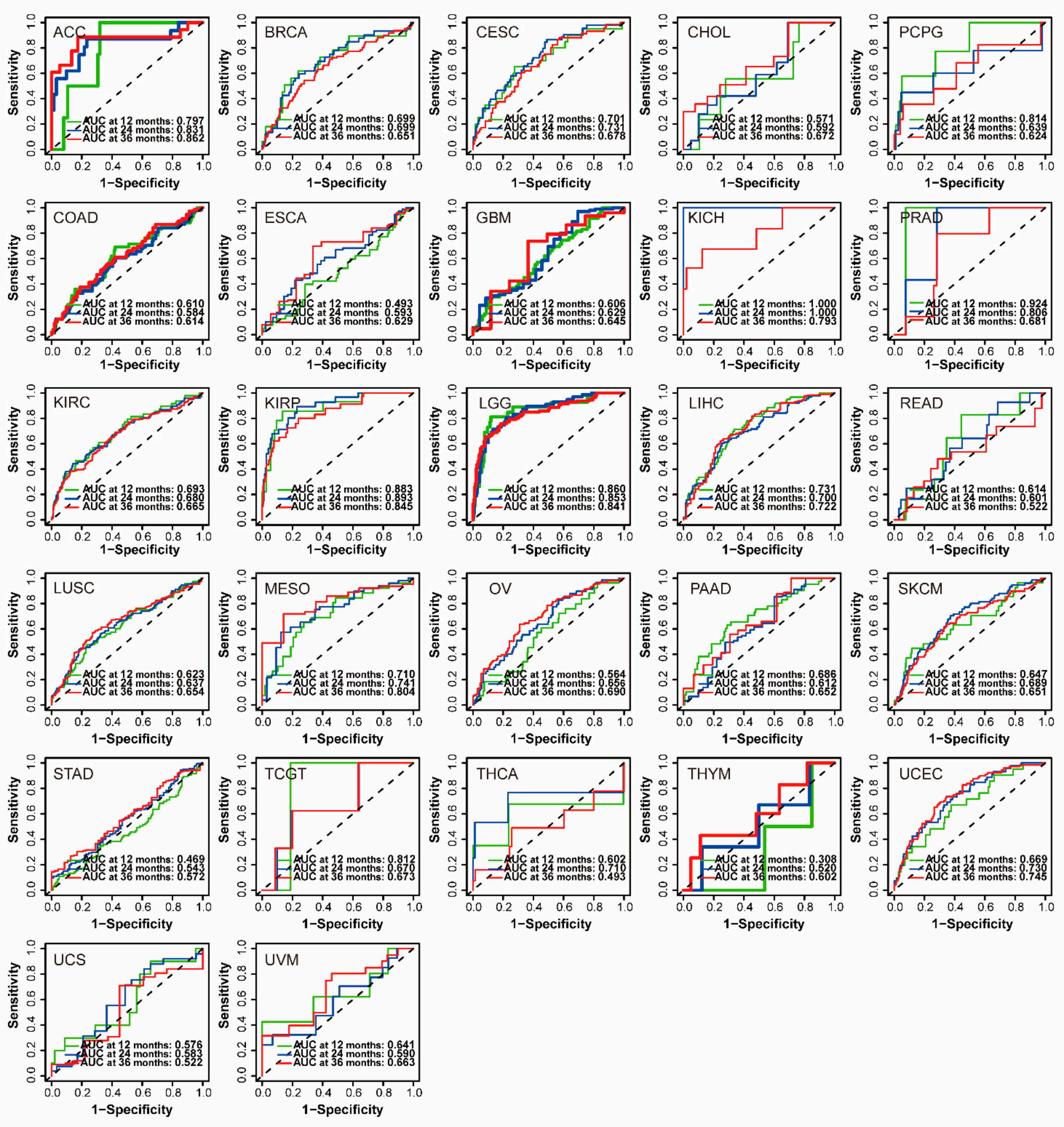

Supplementary Figure S2. ROC analysis of survival-predictive performance of PAGscore in different human cancers. Abbreviations: ACC, drenocortical carcinoma; BRCA, breast invasive carcinoma; CESC, cervical squamous cell carcinoma and endocervical adenocarcinoma; CHOL, cholangiocarcinoma; PCPG, pheochromocytoma and paraganglioma; COAD, colon adenocarcinoma; ESCA, esophageal carcinoma; GBM, glioblastoma multiforme; KICH, kidney chromophobe; PRAD, prostate adenocarcinoma;

KIRC, kidney renal clear cell carcinoma; KIRP, kidney renal papillary cell carcinoma; LGG, brain lower grade glioma; LIHC, liver hepatocellular carcinoma; READ, rectum adenocarcinoma; LUSC, lung squamous cell carcinoma; MESO, mesothelioma; OV, ovarian serous cystadenocarcinoma; PAAD, pancreatic adenocarcinoma; SKCM, skin cutaneous melanoma; STAD, stomach adenocarcinoma; TCGT, testicular germ cell tumors; THCA, thyroid carcinoma; THYM, thymoma; UCEC, uterine corpus endometrial carcinoma; USC, uterine carcinosarcoma; UVM, uveal melanoma.

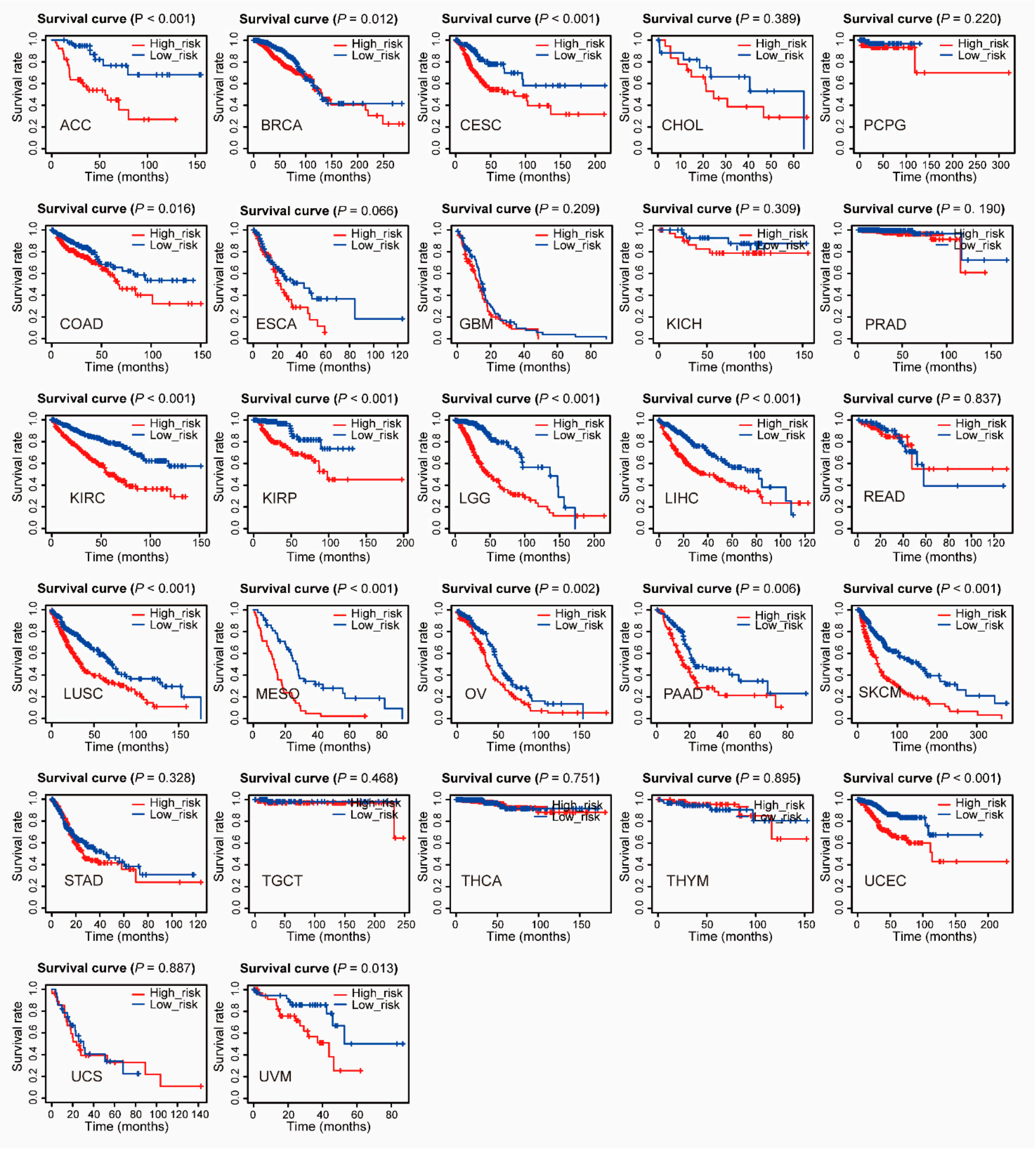

Supplementary Figure S3. Survival analysis of high- and low-risk patients in different human cancers. Abbreviations: ACC, drenocortical carcinoma; BRCA, breast invasive carcinoma; CESC, cervical squamous cell carcinoma and endocervical adenocarcinoma; CHOL, cholangiocarcinoma; PCPG, pheochromocytoma and paraganglioma; COAD, colon adenocarcinoma; ESCA, esophageal carcinoma; GBM, glioblastoma multiforme; KICH,

kidney chromophobe; PRAD, prostate adenocarcinoma; KIRC, kidney renal clear cell carcinoma; KIRP, kidney renal papillary cell carcinoma; LGG, brain lower grade glioma; LIHC, liver hepatocellular carcinoma; READ, rectum adenocarcinoma; LUSC, lung squamous cell carcinoma; MESO, mesothelioma; OV, ovarian serous cystadenocarcinoma; PAAD, pancreatic adenocarcinoma; SKCM, skin cutaneous melanoma; STAD, stomach adenocarcinoma; TCGT, testicular germ cell tumors; THCA, thyroid carcinoma; THYM, thymoma; UCEC, uterine corpus endometrial carcinoma; USC, uterine carcinosarcoma; UVM, uveal melanoma.

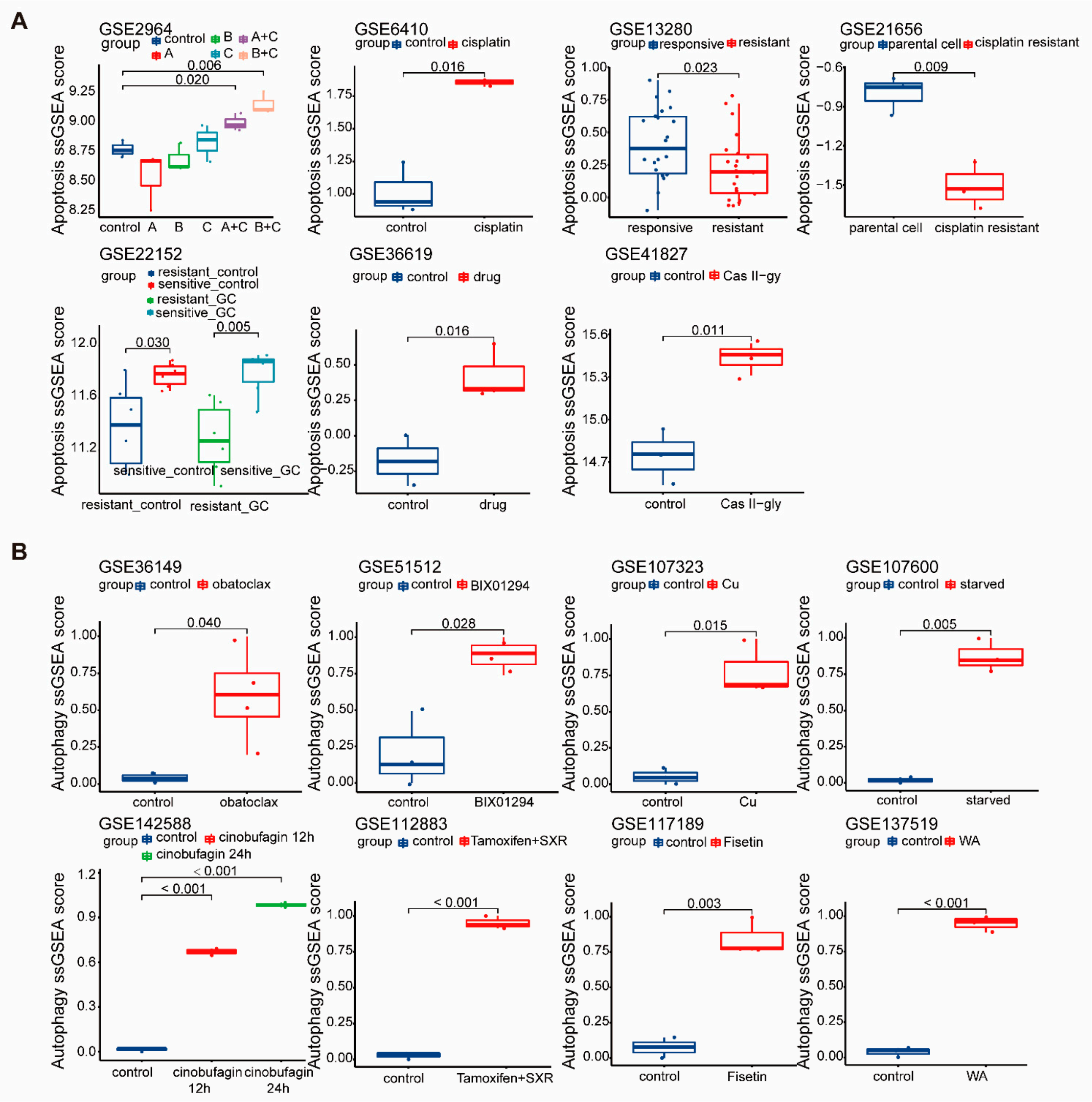

Supplementary Figure S4. Validating the performance of five types cell death gene signatures in differentiating death-inducing and non-death-inducing cells. A) Boxplots showed the signature of apoptosis could effectively differentiate the death-inducing and non-death-inducing cells; B) Boxplots showed the signature of autophagy could effectively differentiate the death-inducing and non-death-inducing cells.

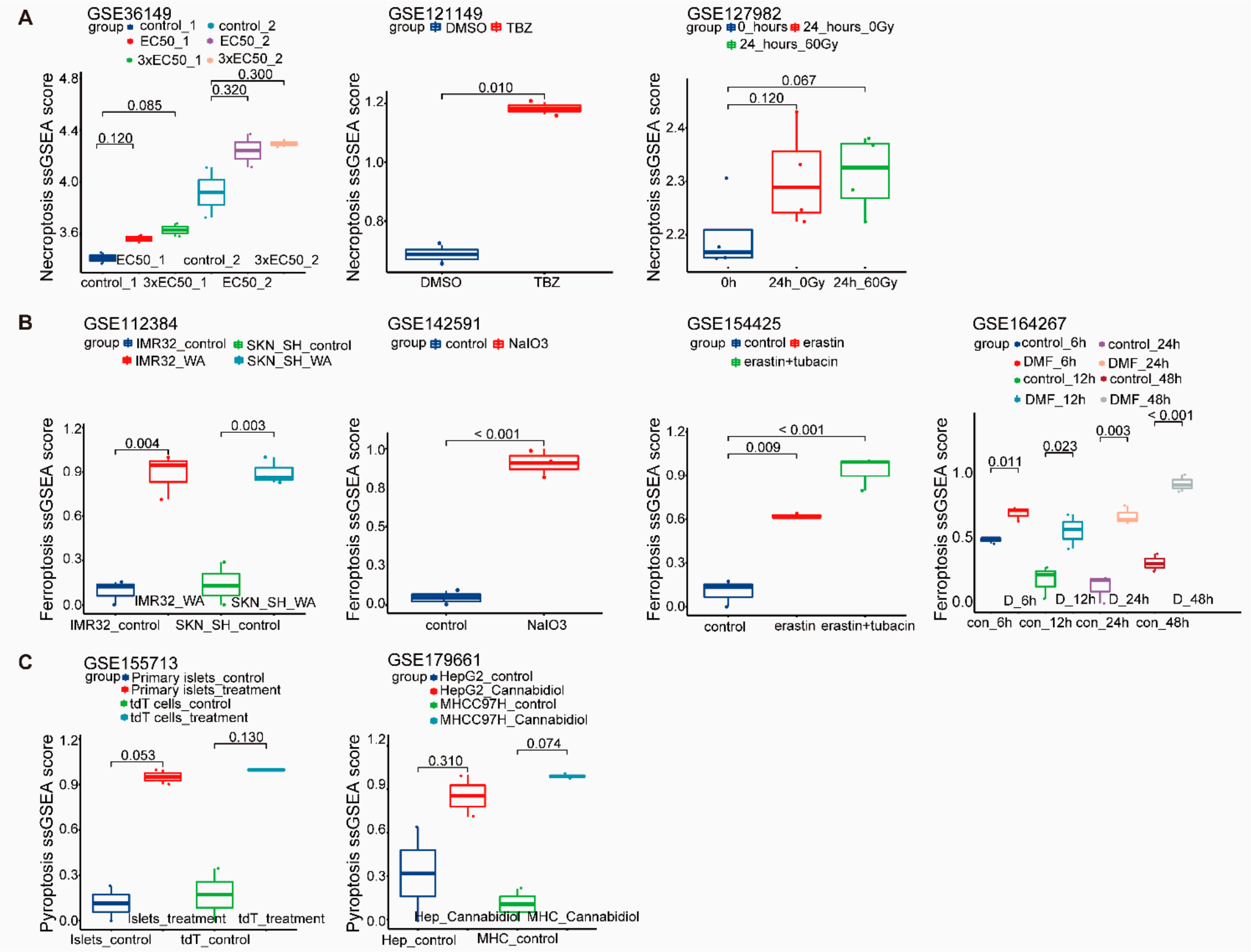

Supplementary Figure S5. Validating the performance of five types cell death gene signatures in differentiating death-inducing and non-death-inducing cells. A) Boxplots showed the signature of necroptosis could potentially differentiate the death-inducing and non-death-inducing cells; B) Boxplots showed the signature of ferroptosis could effectively differentiate the death-inducing and non-death-inducing cells; C) Boxplots showed the signature of pyroptosis could potentially differentiate the death-inducing and non-death-inducing cells.

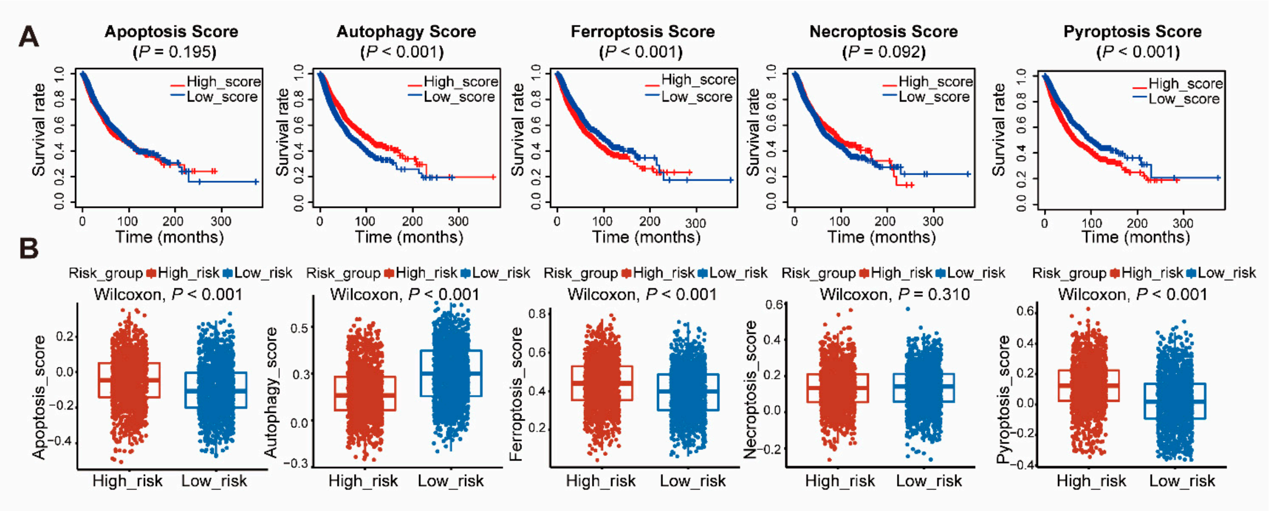

Supplementary Figure S6. Identifying and characterizing five types of cell death gene signatures in cancers. A) Survival analysis showed ferroptosis score and pyroptosis were unfavorable survival predictors, but autophagy score was favorable predictors in validation cohort; B) Box plots showed high-risk patients have higher score of apoptosis, ferroptosis and pyroptosis, and lower score of autophagy than low-risk patients.

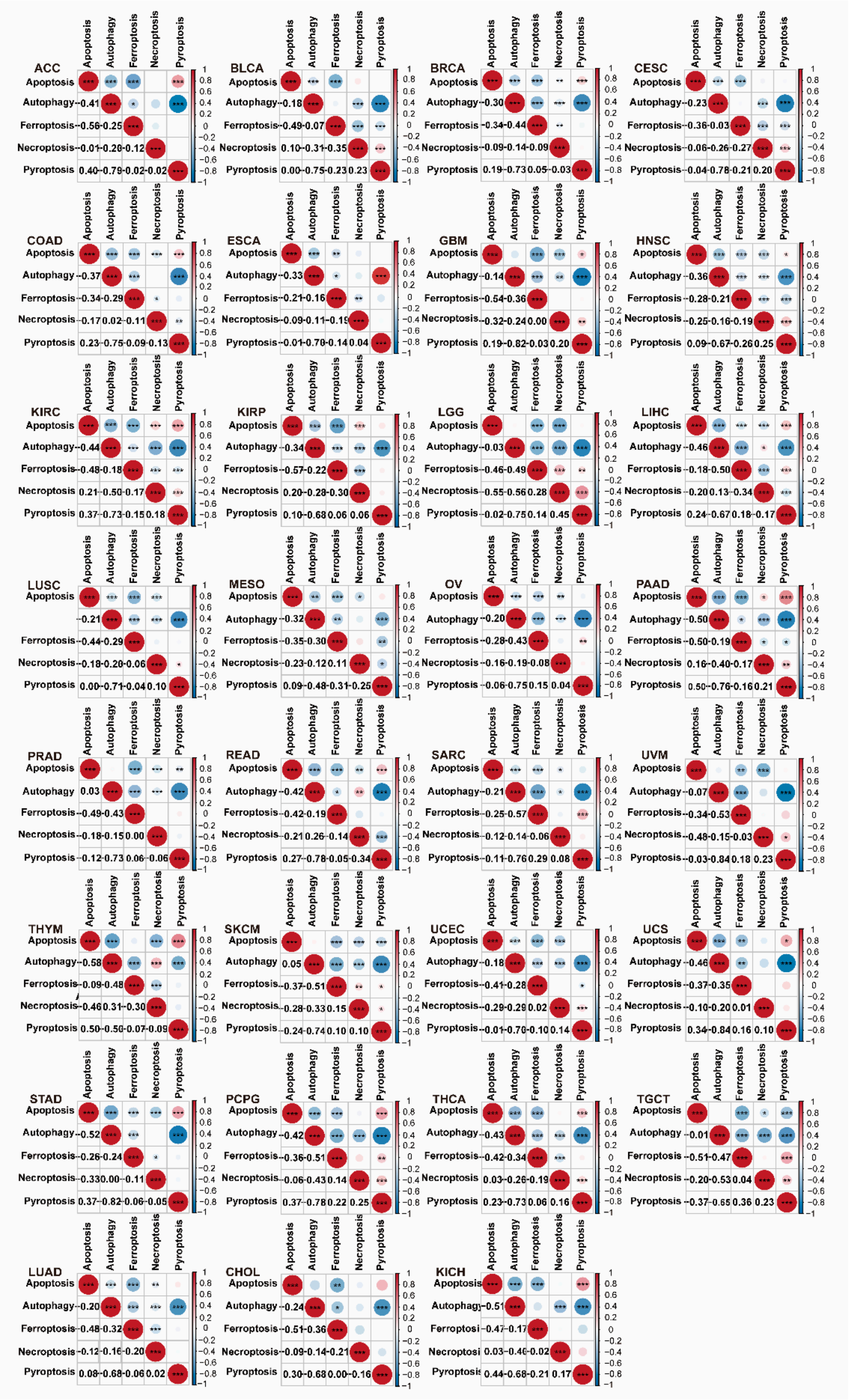

Supplementary Figure S7. Correlation analysis of score of five types of cell death signatures in different types of cancers. Abbreviations: ACC, drenocortical carcinoma; BRCA, breast invasive carcinoma; CESC, cervical squamous cell carcinoma and endocervical adenocarcinoma; CHOL, cholangiocarcinoma; PCPG, pheochromocytoma and paraganglioma; COAD, colon adenocarcinoma; ESCA, esophageal carcinoma; GBM, glioblastoma multiforme; KICH, kidney chromophobe; PRAD, prostate adenocarcinoma; KIRC, kidney renal clear cell carcinoma; KIRP, kidney renal papillary cell carcinoma; LGG, brain lower grade glioma; LIHC, liver hepatocellular carcinoma; READ, rectum adenocarcinoma; LUSC, lung squamous cell carcinoma; MESO, mesothelioma; OV, ovarian serous cystadenocarcinoma; PAAD, pancreatic adenocarcinoma; SKCM, skin cutaneous melanoma; STAD, stomach adenocarcinoma; TCGT, testicular germ cell tumors; THCA, thyroid carcinoma; THYM, thymoma; UCEC, uterine corpus endometrial carcinoma; USC, uterine carcinosarcoma; UVM, uveal melanoma. *: $P < 0.05$, **: $P < 0.01$, ***: $P < 0.001$.

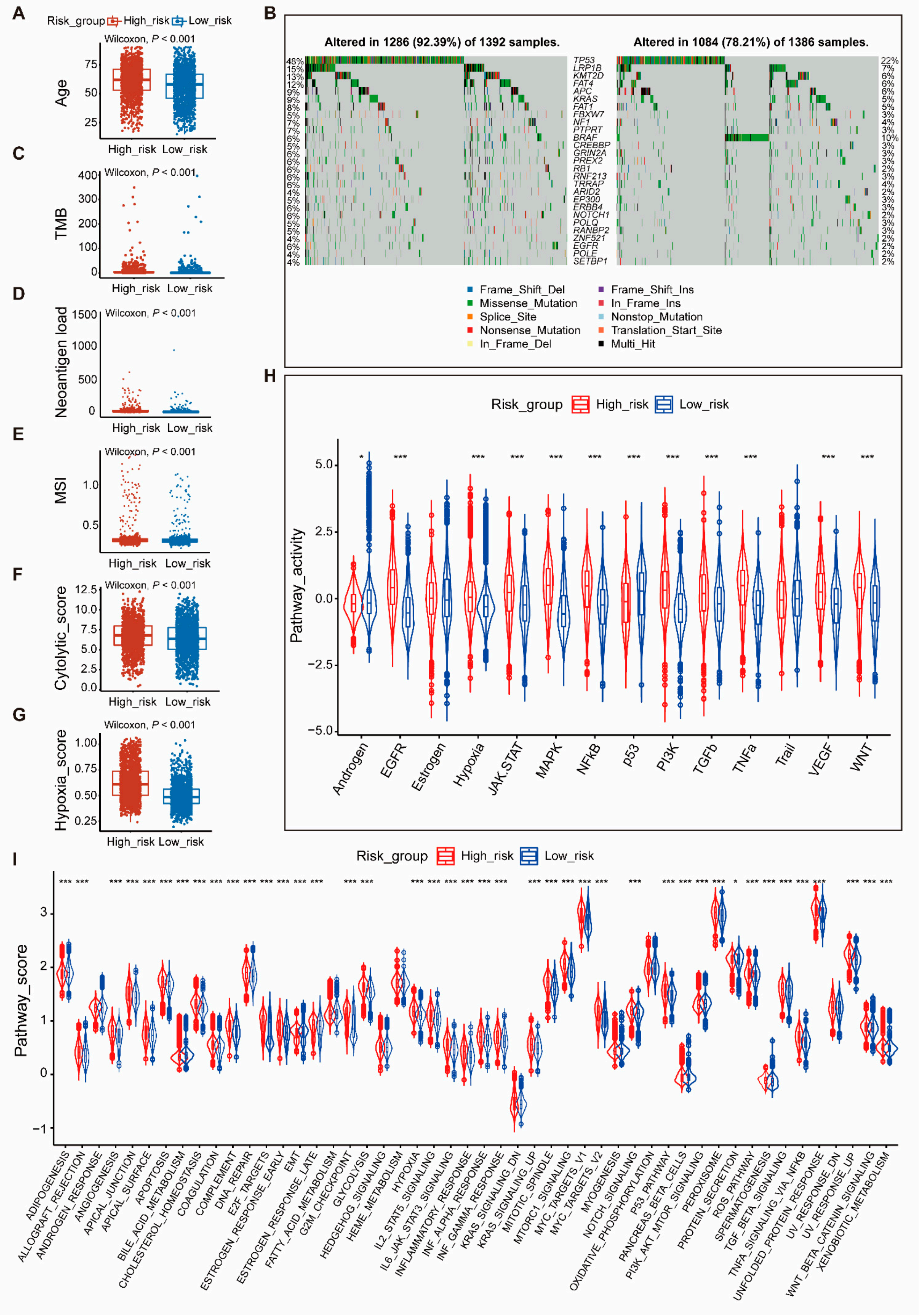

A
Risk_group High_risk Low_risk
Wilcoxon, P < 0.001
Age
High_risk Low_risk
B
Altered in 1286 (92.39%) of 1392 samples.
Altered in 1084 (78.21%) of 1386 samples.
TP53
LRP1B
KMT2D
FAT4
APC
KRAS
FAT1
FBXW7
NF1
PTPRT
BRAF
CREBBP
GRIN2A
PREX2
RB1
RNF213
TRRAP
ARID2
EP300
ERBB4
NOTCH1
POLQ
RANBP2
ZNF521
EGFR
POLE
SETBP1
Frame_Shift_Del
Missense_Mutation
Splice_Site
Nonsense_Mutation
In_Frame_Del
Frame_Shift_Ins
In_Frame_Ins
Nonstop_Mutation
Translation_Start_Site
Multi_Hit
C
TMB
D
Neoantigen load
E
MSI
F
Cytolytic_score
G
Hypoxia_score
H
Pathway_activity
Androgen
EGFR
Estrogen
Hypoxia
JAK.STAT
MAPK
NFkB
p53
PI3K
TGFb
TNFa
Trail
VEGF
WNT
I
Pathway_score
ADIPOGENESIS
ALLOGRAFT_REJECTION
ANDROGEN_RESPONSE
ANGIOGENESIS
APICAL_JUNCTION
APICAL_SURFACE
APOPTOSIS
BILE_ACID_METABOLISM
CHOLESTEROL_HOMEOSTASIS
COAGULATION
COMPLEMENT
DNA_REPAIR
E2F_TARGETS
ESTROGEN_RESPONSE_EARLY
EMT
ESTROGEN_RESPONSE_LATE
FATTY_ACID_METABOLISM
G2M_CHECKPOINT
GLYCOLYSIS
HEDGEHOG_SIGNALING
HEME_METABOLISM
HYPOXIA
IL2_STAT5_SIGNALING
IL6_JAK_STAT3_SIGNALING
INFLAMMATORY_RESPONSE
INF_ALPHA_RESPONSE
INF_GAMMA_RESPONSE
KRAS_SIGNALING_DN
KRAS_SIGNALING_UP
MITOTIC_SPINDLE
MTORC1_SIGNALING
MYC_TARGETS_V1
MYC_TARGETS_V2
MYOGENESIS
NOTCH_SIGNALING
OXIDATIVE_PHOSPHORYLATION
P53_PATHWAY
PANCREAS_BETA_CELLS
PI3K_AKT_MTOR_SIGNALING
PEROXISOME
PROTEIN_SECRETION
ROS_PATHWAY
SPERMATOGENESIS
TGF_BETA_SIGNALING
TNFA_SIGNALING_VIA_NFKB
UNFOLDED_PROTEIN_RESPONSE
UV_RESPONSE_DN
UV_RESPONSE_UP
WNT_BETA_CATENIN_SIGNALING
XENOBIOTIC_METABOLISM

Supplementary Figure S8. Comparing the clinical and molecular features between high- and low- risk patients in validation cohort. A) Box plot showed high-risk patients have older-age distribution than low-risk patients; B) Analysis of the mutation profiles of the fifty most commonly mutated genes in human cancers showed 23 genes had significantly different mutation frequency between low-risk and high-risk patients ; C) Box plot showed high-risk patients have higher TMB than low-risk patients; D) Box plot showed high-risk patients have higher cytolytic score than low-risk patients; E) Box plot showed high-risk patients have higher hypoxia score than low-risk patients; F) Relative signaling pathway activity scores in high- and low- risk patients measured from RNA-seq by progeny; G) Violin plot showed the ssGSEA score of fifty pathways of cancer hallmarkers in high- and low-risk patients. Abbreviations: TMB, tumor mutation burden. *: $P < 0.05$, **: $P < 0.01$, ***: $P < 0.001$.

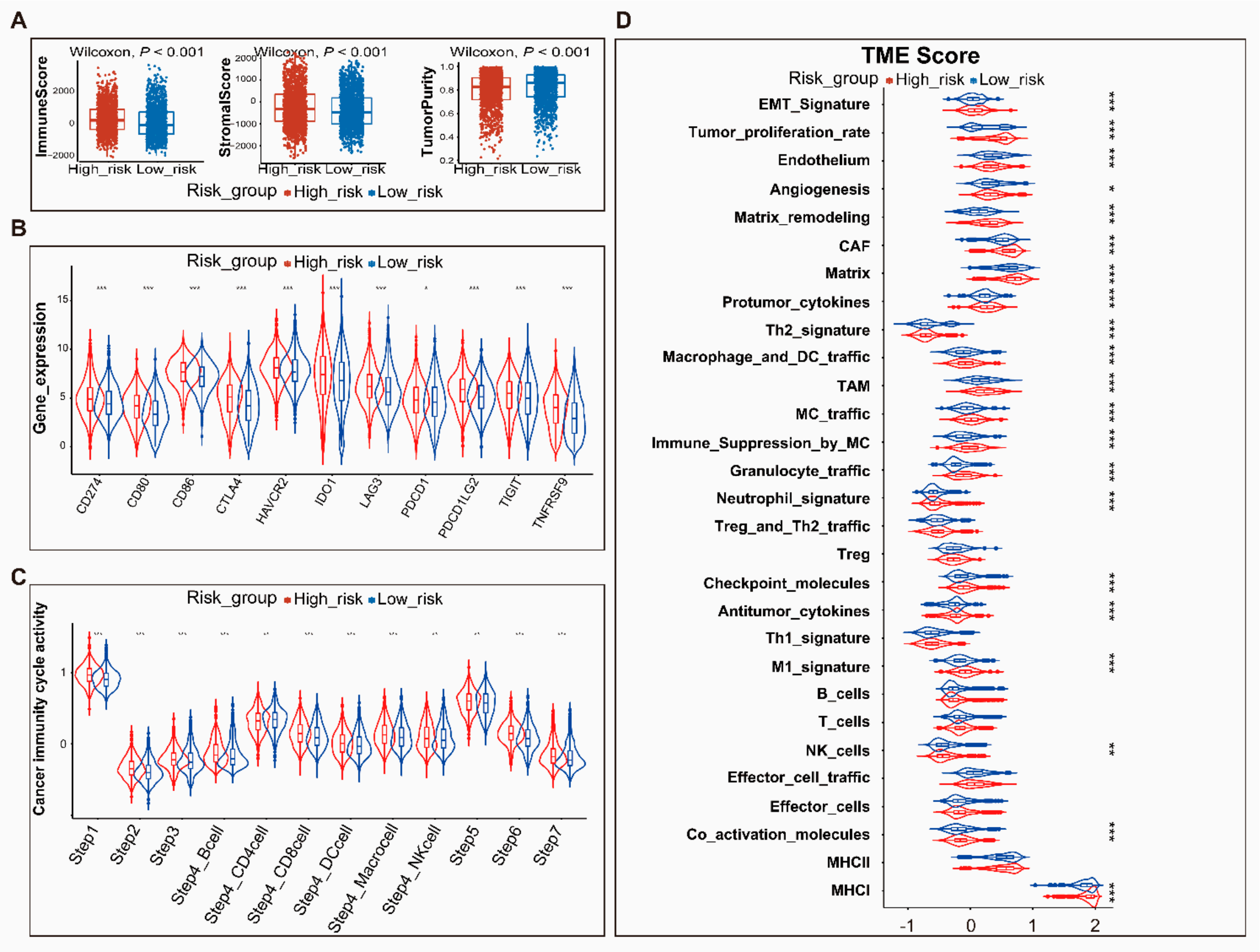

Supplementary Figure S9. Comparing the differences of tumor microenvironment between high- and low- risk patients in validation cohort. A) Box plots showed high-risk patients have higher “ImmuneScore” and “StromaScore”, and lower “TumorPurity” than low-risk patients; B) The violin plot showed the expression of immune checkpoint genes in high- and low-risk patients; C) The relative activity of seven-step of cancer immunity cycle in high- and low-risk patients.; D) The violin plot showed the ssGSEA score of 29 components of tumor microenvironment in high- and low-risk patients. Abbreviations: CAF, cancer associated fibroblasts; TAM, tumor associated macrophages; MC, myeloid cells. *: $P < 0.05$, **: $P < 0.01$, ***: $P < 0.001$.

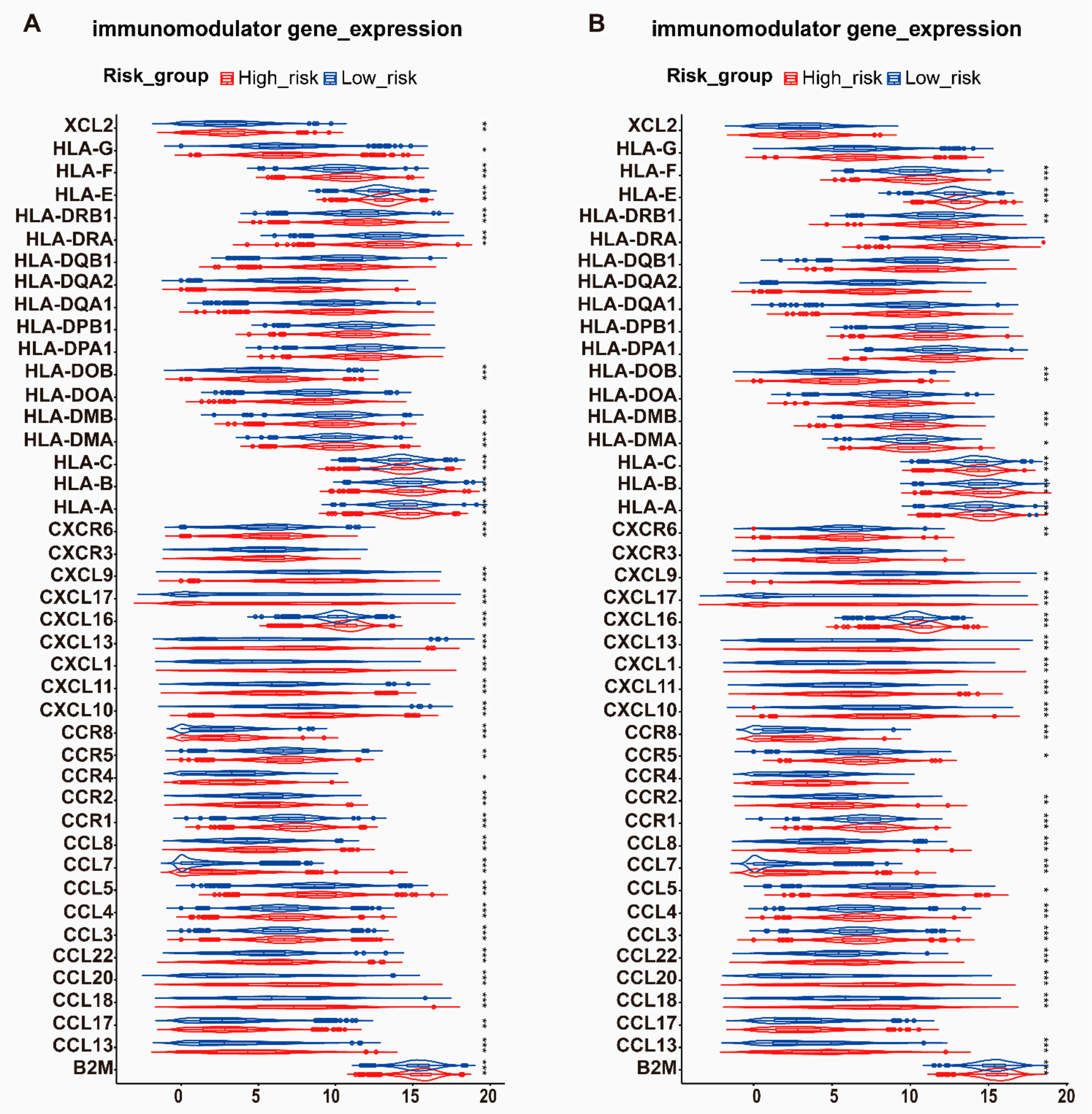

Supplementary Figure S10. The expression of immune-related genes in high- and low-risk patients. A) The expression of immune-related genes in high- and low-risk patients in training cohort; B) The expression of immune-related genes in high- and low-risk patients in validation cohort. *: $P < 0.05$, **: $P < 0.01$, ***: $P < 0.001$.

1.2 Supplementary Table

Supplementary Table S1. Univariate Cox regression analysis for overall survival in training cohort

Gene	HR	HR.95 CI Lower	HR.95 CI Upper	P value
BIRC5	1.21	1.18	1.24	0.000
PSMD2	1.58	1.49	1.67	0.000
TFRC	1.24	1.21	1.28	0.000
IL1A	1.12	1.1	1.13	0.000
PSMD14	1.78	1.65	1.91	0.000
CLSPN	1.19	1.17	1.22	0.000
BCL2	0.83	0.81	0.85	0.000
TUBA1B	1.51	1.42	1.59	0.000
SLC7A11	1.15	1.13	1.18	0.000
PSMA7	1.55	1.46	1.65	0.000
BAK1	1.4	1.33	1.46	0.000
PSMB2	1.64	1.53	1.76	0.000
CASP4	1.3	1.25	1.35	0.000
ACTB	1.6	1.49	1.71	0.000
PIK3R3	0.79	0.77	0.82	0.000
NRAS	1.44	1.37	1.53	0.000
PYGL	1.19	1.16	1.22	0.000
PLEC	1.34	1.29	1.41	0.000

LMNB1	1.22	1.18	1.26	0.000
PSMD11	1.46	1.37	1.54	0.000
STEAP3	1.21	1.18	1.25	0.000
LMNB2	1.29	1.24	1.35	0.000
TUBA1C	1.24	1.19	1.28	0.000
E2F1	1.22	1.18	1.26	0.000
PSMD7	1.59	1.47	1.71	0.000
DYNLL1	1.58	1.46	1.7	0.000
PSMC2	1.66	1.53	1.8	0.000
YWHAG	1.48	1.38	1.58	0.000
IL1B	1.13	1.11	1.15	0.000
BID	1.31	1.25	1.37	0.000
TNFRSF1A	1.43	1.34	1.52	0.000
TUBA3D	0.87	0.85	0.89	0.000
PGAM5	1.37	1.3	1.45	0.000
TNFRSF21	1.19	1.15	1.23	0.000
TUBB6	1.21	1.17	1.26	0.000
TUBB2A	1.15	1.12	1.18	0.000
PSMD1	1.56	1.44	1.69	0.000
TUBB3	1.14	1.11	1.17	0.000
IFNAR2	1.36	1.29	1.44	0.000
OMA1	0.75	0.71	0.79	0.000

MYC	1.17	1.14	1.21	0.000
PSMA1	1.58	1.45	1.72	0.000
TRAF3	1.37	1.29	1.46	0.000
YWHAZ	1.33	1.26	1.41	0.000
PSMC4	1.42	1.33	1.52	0.000
USP30	0.7	0.66	0.75	0.000
PSME3	1.52	1.4	1.65	0.000
IFT88	0.79	0.75	0.83	0.000
PPIA	1.43	1.34	1.54	0.000
SATB1	0.87	0.85	0.89	0.000
FADD	1.3	1.23	1.36	0.000
STAT5B	0.73	0.69	0.78	0.000
TUBA3E	0.88	0.86	0.9	0.000
CASP1	1.16	1.13	1.19	0.000
CASP5	1.12	1.09	1.14	0.000
TFDP1	1.3	1.23	1.37	0.000
PLA2G4E	1.08	1.06	1.1	0.000
ITPR1	0.87	0.85	0.9	0.000
CAPN2	1.29	1.23	1.36	0.000
PSMD12	1.37	1.29	1.46	0.000
EIF2AK2	1.26	1.2	1.32	0.000

CASP3	1.37	1.28	1.46	0.000
SLC25A5	1.25	1.2	1.31	0.000
YWHAQ	1.42	1.32	1.53	0.000
CAMK2B	0.93	0.92	0.94	0.000
PLA2G4A	1.11	1.09	1.14	0.000
UBC	1.45	1.34	1.56	0.000
IGF1R	0.88	0.86	0.91	0.000
ATG2B	0.75	0.71	0.8	0.000
PSMA5	1.35	1.27	1.44	0.000
PSMA2	1.4	1.3	1.5	0.000
PPP1R13B	0.81	0.77	0.84	0.000
FTL	1.19	1.15	1.24	0.000
LY96	1.14	1.11	1.17	0.000
CAMK2A	1.07	1.06	1.09	0.000
VDAC1	1.35	1.26	1.44	0.000
IL18	1.12	1.09	1.15	0.000
PPP3R1	1.44	1.33	1.57	0.000
GCLM	1.17	1.13	1.21	0.000
PAK2	1.41	1.3	1.52	0.000
CTSO	0.85	0.82	0.88	0.000
DYNLL2	0.79	0.75	0.83	0.000
EIF2S1	1.41	1.31	1.53	0.000

CTSC	1.14	1.11	1.17	0.000
STAT2	1.29	1.22	1.37	0.000
CYCS	1.25	1.18	1.31	0.000
YWHAB	1.43	1.32	1.56	0.000
DAPK2	0.9	0.88	0.92	0.000
GPX4	0.8	0.76	0.84	0.000
AVEN	1.29	1.22	1.37	0.000
NBR1	0.74	0.69	0.79	0.000
IGBP1	0.74	0.69	0.8	0.000
RRAGC	1.23	1.17	1.29	0.000
PLIN3	1.26	1.19	1.33	0.000
TNFAIP3	1.15	1.11	1.19	0.000
GABARAP	0.76	0.71	0.81	0.000
PSMF1	1.52	1.38	1.68	0.000
RRAGB	0.81	0.77	0.85	0.000
ATG9A	1.42	1.3	1.54	0.000
TOMM5	1.29	1.21	1.37	0.000
ATG12	1.44	1.32	1.58	0.000
LMNA	1.19	1.14	1.24	0.000
GADD45A	1.17	1.13	1.22	0.000
MAP3K1	0.85	0.82	0.89	0.000

RNASE1	1.12	1.09	1.15	0.000
TSC2	0.75	0.7	0.8	0.000
HELLS	1.12	1.09	1.15	0.000
VPS37C	0.75	0.7	0.81	0.000
CSNK2A1	1.36	1.26	1.46	0.000
CP	1.04	1.03	1.06	0.000
GADD45G	0.92	0.9	0.94	0.000
MFN1	1.25	1.18	1.32	0.000
RELA	1.45	1.32	1.59	0.000
TNFRSF10B	1.18	1.13	1.24	0.000
BCL2A1	1.09	1.06	1.11	0.000
PIK3R1	0.87	0.84	0.9	0.000
PYCARD	1.12	1.09	1.16	0.000
PSMB5	1.33	1.24	1.44	0.000
PRKCD	0.87	0.84	0.9	0.000
TOMM40	1.24	1.17	1.31	0.000
UBE2V1	1.42	1.3	1.56	0.000
MAPK9	0.76	0.71	0.82	0.000
MAP2K1	1.33	1.23	1.43	0.000
TSC1	0.76	0.71	0.82	0.000
BMF	0.88	0.85	0.91	0.000
PLA2G4C	0.91	0.88	0.93	0.000

SLC25A4	0.87	0.83	0.9	0.000
CYLD	0.82	0.77	0.86	0.000
IKBKG	1.26	1.19	1.34	0.000
DSG1	1.05	1.03	1.06	0.000
CD14	1.11	1.08	1.15	0.000
SFN	1.04	1.03	1.05	0.000
PSMD8	1.31	1.22	1.41	0.000
PSMB9	1.12	1.08	1.15	0.000
IL3RA	0.88	0.85	0.91	0.000
ITPR2	0.89	0.86	0.92	0.000
IGF2	1.05	1.04	1.06	0.000
AKT3	0.92	0.89	0.94	0.000
KPNA1	1.38	1.26	1.51	0.000
MAP2K4	0.79	0.74	0.84	0.000
EEF1A1	0.8	0.76	0.86	0.000
SLC39A14	1.12	1.09	1.16	0.000
PIK3CA	1.19	1.13	1.25	0.000
C1QBP	1.23	1.16	1.3	0.000
PPP3CC	0.82	0.77	0.86	0.000
HSPA8	1.24	1.17	1.32	0.000
MTMR3	0.79	0.73	0.84	0.000

MAPT	0.95	0.94	0.96	0.000
SLC3A2	1.19	1.14	1.25	0.000
MCL1	1.25	1.17	1.34	0.000
PSMD3	1.23	1.16	1.3	0.000
CAPN1	1.2	1.14	1.27	0.000
ACSL4	1.14	1.1	1.18	0.000
DFFA	1.29	1.2	1.39	0.000
CHMP7	0.75	0.69	0.82	0.000
TUBA4A	1.09	1.07	1.12	0.000
CASP10	1.12	1.09	1.16	0.000
APIP	0.81	0.76	0.86	0.000
STK24	1.23	1.16	1.31	0.000
CASP12	0.88	0.85	0.92	0.000
DSG3	1.03	1.02	1.04	0.000
PRKAB2	1.17	1.12	1.22	0.000
MAP1LC3B2	1.25	1.17	1.34	0.000
ATG4A	0.8	0.75	0.86	0.000
NCOA4	0.81	0.76	0.86	0.000
APPL1	0.8	0.75	0.86	0.000
PSMD4	1.24	1.16	1.33	0.000
TICAM1	1.2	1.14	1.27	0.000
TUBB8	0.9	0.87	0.93	0.000

PRNP	1.12	1.08	1.16	0.000
PSMB3	1.2	1.13	1.27	0.000
ACTG1	1.25	1.17	1.34	0.000
NFKBIB	1.21	1.14	1.29	0.000
PSMC3	1.29	1.19	1.39	0.000
SMPD1	0.86	0.83	0.91	0.000
CASP8	1.16	1.1	1.21	0.000
DBNL	1.28	1.18	1.38	0.000
HMOX1	1.09	1.06	1.12	0.000
PDPK1	0.84	0.8	0.89	0.000
BCL2L2	0.85	0.8	0.89	0.000
SLC11A2	0.83	0.78	0.88	0.000
KPNB1	1.28	1.18	1.38	0.000
SRC	1.17	1.11	1.22	0.000
TOMM20	0.8	0.75	0.86	0.000
SLC25A6	0.86	0.81	0.9	0.000
PRKAG2	0.88	0.84	0.92	0.000
STAT1	1.12	1.08	1.16	0.000
PRKAA2	0.95	0.94	0.97	0.000
CHMP4B	1.27	1.17	1.37	0.000
ULK2	0.88	0.85	0.92	0.000

ATG10	0.81	0.76	0.87	0.000
SLC38A9	1.22	1.14	1.3	0.000
YWHAH	1.21	1.14	1.29	0.000
TP73	1.06	1.04	1.08	0.000
MLKL	1.12	1.08	1.16	0.000
CAMK2G	1.16	1.1	1.22	0.000
NFKBIE	1.15	1.1	1.21	0.000
GLUD1	0.86	0.82	0.91	0.000
GCLC	1.11	1.07	1.15	0.000
PINK1	0.86	0.82	0.91	0.000
RBCK1	1.21	1.13	1.29	0.000
AMBRA1	0.78	0.72	0.85	0.000
ADD1	0.8	0.74	0.87	0.000
UACA	0.89	0.85	0.93	0.000
TUBB2B	1.04	1.03	1.05	0.000
CASP2	1.18	1.11	1.25	0.000
XIAP	0.82	0.76	0.88	0.000
PSMA6	1.19	1.12	1.26	0.000
MAP1LC3C	1.07	1.04	1.09	0.000
IRF3	1.17	1.11	1.24	0.000
PSMD13	1.24	1.15	1.34	0.000
GFAP	1.03	1.02	1.04	0.000

TUBA1A	1.09	1.06	1.12	0.000
PLA2G4F	0.97	0.95	0.98	0.000
MAPK10	0.95	0.93	0.97	0.000
VDAC2	1.21	1.13	1.3	0.000
IRF6	0.96	0.95	0.98	0.000
TNFRSF10A	1.08	1.05	1.11	0.000
TP53AIP1	1.05	1.03	1.07	0.000
DYNC1LI1	0.8	0.73	0.87	0.000
NMT1	1.32	1.19	1.47	0.000
PARP1	1.16	1.1	1.23	0.000
BIRC2	1.19	1.11	1.27	0.000
GABARAPL1	0.91	0.88	0.94	0.000
BMX	0.94	0.92	0.96	0.000
CFLAR	1.14	1.08	1.2	0.000
PSMA4	1.2	1.12	1.28	0.000
CDKN2A	1.04	1.03	1.06	0.000
IRF5	1.1	1.06	1.14	0.000
MLST8	0.84	0.78	0.9	0.000
TNFRSF25	1.07	1.04	1.1	0.000
CTSF	0.93	0.9	0.96	0.000
SLC40A1	0.94	0.92	0.96	0.000

CTSK	1.05	1.03	1.08	0.000
IFNGR1	1.14	1.08	1.2	0.000
SAT2	0.89	0.85	0.94	0.000
HDAC6	0.84	0.79	0.9	0.000
PMAIP1	1.06	1.04	1.09	0.000
DCC	1.05	1.03	1.07	0.000
JMJD7-PLA2G4B	0.9	0.86	0.94	0.000
PSMB8	1.1	1.06	1.15	0.000
TUBA3C	0.93	0.9	0.96	0.000
HMGB2	1.1	1.05	1.14	0.000
MTMR14	0.82	0.76	0.89	0.000
PSMD10	1.2	1.11	1.3	0.000
OPA1	1.18	1.1	1.27	0.000
PIK3CB	0.88	0.83	0.93	0.000
FTMT	0.69	0.59	0.81	0.000
HSF1	1.17	1.09	1.25	0.000
HRAS	1.12	1.07	1.17	0.000
FUNDC1	0.85	0.8	0.91	0.000
GABARAPL2	0.86	0.8	0.92	0.000
UNC5B	1.07	1.04	1.11	0.000
KRAS	1.13	1.07	1.2	0.000
STAT3	1.17	1.09	1.26	0.000

DAPK3	1.15	1.08	1.22	0.000
HSP90AA1	1.14	1.08	1.21	0.000
IRF1	1.08	1.04	1.12	0.000
PSMB4	1.16	1.09	1.24	0.000
ZBP1	1.05	1.03	1.07	0.000
FTH1	1.12	1.06	1.18	0.000
IFNAR1	0.84	0.77	0.91	0.000
TNF	1.05	1.03	1.07	0.000
ACIN1	0.83	0.76	0.91	0.000
IRF7	1.07	1.04	1.11	0.000
PARK7	0.84	0.78	0.91	0.000
IFNGR2	1.15	1.07	1.22	0.000
BIRC3	1.05	1.02	1.07	0.000
PSMB7	1.15	1.08	1.24	0.000
HTRA2	1.2	1.1	1.31	0.000
ATM	0.9	0.86	0.95	0.000
PARP2	1.15	1.08	1.22	0.000
TRPM7	0.88	0.83	0.94	0.000
PRKAB1	0.89	0.84	0.94	0.000
EIF2AK3	0.89	0.84	0.94	0.000
IGF1	0.96	0.94	0.98	0.000

CARD8	0.89	0.84	0.94	0.000
TF	1.02	1.01	1.03	0.000
VPS37D	0.95	0.93	0.97	0.000
RRAGD	0.95	0.92	0.97	0.000
RIPK3	1.05	1.03	1.08	0.000
FNTA	1.16	1.08	1.25	0.000
PRKAG3	1.07	1.03	1.1	0.000
IFNA13	1.17	1.08	1.27	0.000
STAT4	0.95	0.93	0.98	0.000
TLR4	0.94	0.91	0.97	0.000
OCLN	0.96	0.95	0.98	0.000
RNF31	0.85	0.78	0.92	0.000
UBAP1	1.18	1.08	1.28	0.000
MAP3K5	0.94	0.91	0.97	0.000
PRKCQ	0.96	0.94	0.98	0.000
CHMP6	0.88	0.83	0.94	0.000
ATG16L1	0.87	0.81	0.93	0.000
CHMP4C	0.97	0.96	0.99	0.000
BCAP31	1.14	1.06	1.22	0.000
PSME4	1.12	1.05	1.18	0.000
CHMP1B	1.15	1.07	1.23	0.000
TP53BP2	1.11	1.05	1.18	0.000

CETN1	0.77	0.67	0.89	0.000
TRADD	1.09	1.04	1.14	0.000
ATG4D	0.9	0.85	0.95	0.000
TUBAL3	1.04	1.02	1.06	0.000
PSMB1	1.14	1.06	1.22	0.000
BBC3	0.93	0.89	0.97	0.000
RHEB	1.13	1.06	1.22	0.000
CAMK2D	1.09	1.04	1.15	0.000
STAT6	0.93	0.89	0.97	0.000
TRAF5	1.06	1.03	1.1	0.000
DAXX	1.17	1.07	1.27	0.000
CRADD	0.91	0.86	0.96	0.000
CSNK2B	1.15	1.06	1.24	0.000
ATG9B	1.04	1.02	1.06	0.001
PSMA8	0.92	0.87	0.96	0.001
CYBB	1.05	1.02	1.07	0.001
ACSL3	0.92	0.87	0.96	0.001
VPS37B	1.1	1.04	1.16	0.001
JAK3	1.05	1.02	1.08	0.001
AKT1S1	1.14	1.06	1.23	0.001
PTPN13	0.96	0.94	0.99	0.001

DAPK1	1.05	1.02	1.08	0.001
PSMA3	1.13	1.05	1.21	0.001
IFNB1	1.12	1.05	1.19	0.001
RRAGA	0.88	0.82	0.95	0.001
PSMB6	1.13	1.05	1.21	0.001

Abbreviations: HR, hazard ratio; CI, confidential interval.
